# Social Housing Improves Dairy Calves' Performance in Two Cognitive Tests

**DOI:** 10.1371/journal.pone.0090205

**Published:** 2014-02-26

**Authors:** Charlotte Gaillard, Rebecca K. Meagher, Marina A. G. von Keyserlingk, Daniel M. Weary

**Affiliations:** Animal Welfare Program, Faculty of Land and Food Systems, University of British Columbia, Vancouver, British Columbia, Canada; Queen Mary, University of London, United Kingdom

## Abstract

Early social housing is known to benefit cognitive development in laboratory animals. Pre-weaned dairy calves are typically separated from their dam immediately after birth and housed alone, but no work to date has addressed the effect of individual housing on cognitive performance of these animals. The aim of this study was to determine the effects of individual versus social housing on two measures of cognitive performance: reversal learning and novel object recognition. Holstein calves were either housed individually in a standard calf pen (n = 8) or kept in pairs using a double pen (n = 10). Calves were tested twice daily in a Y-maze starting at 3 weeks of age. Calves were initially trained to discriminate two colours (black and white) until they reached a learning criterion of 80% correct over three consecutive sessions. Training stimuli were then reversed (i.e. the previously rewarded colour was now unrewarded, and vice-versa). Calves from the two treatments showed similar rates of learning in the initial discrimination task, but the individually housed calves showed poorer performance in the reversal task. At 7 weeks of age, calves were tested for their response to a novel object in eight tests over a two-day period. Pair-housed calves showed declining exploration with repeated testing but individually reared calves did not. The results of these experiments provide the first direct evidence that individual housing impairs cognitive performance in dairy calves.

## Introduction

On many dairy farms calves are separated from the cow and housed in individual pens until they are weaned from milk at approximately 6 to 8 weeks of age. Individual housing is preferred by some farmers on the basis of ease of management and perceived benefits to calf health, but the practice has been criticized on welfare grounds as it limits the opportunity for the calves to perform social behaviours [1], [2]. Social housing early in life is known to benefit calves by reducing weaning distress and improving performance after weaning when calves are typically introduced into group housing [3]–[5].

Early social housing may also benefit cognitive development. Laboratory animals reared in partial or complete social isolation show cognitive deficits relative to socially reared counterparts [6], [7]. For example, individually reared rats typically show similar performance to socially housed rats in simple discrimination tasks, but are slower to learn when test stimuli or rules are changed [8]. One approach to assessing this ability to “re-learn” is to reverse training cues such that the original positive cue becomes negative and vice versa. The Novel Object Recognition test has also been used to study learning and memory [9], taking advantage of the animal's natural tendency to explore novel objects and environments. Recognition of the object is assessed based on habituation of this exploratory response. Rats reared in isolation show impaired performance in this test relative to socially reared counterparts [8].

No work to date has assessed reversal learning, novel object recognition or indeed any direct measure of cognitive performance in differentially housed calves. However, earlier work has shown that individually reared calves take longer to access feed from an unfamiliar feeder [4], [5], suggesting that these animals may have poorer problem solving abilities. More generally, individually reared calves seem to have difficulty in coping with novel situations [3].

The aim of the current study was to test the effects of individual versus pair housing of dairy calves on cognitive performance. Experiment 1 tested the effect of these treatments on initial discrimination learning and reversal learning. We predicted that initial discrimination learning would be similar for individually and socially reared calves, but individually reared calves would show slower learning on the reversal task. Experiment 2 tested the effects of individual versus pair housing on novel object recognition. We predicted that pair-housed calves would habituate to the novel object during repeated exposures, but individually reared calves would not.

## Materials and Methods

### (a) Ethics Statement

This study was approved by the Canadian Council on Animal Care (Protocol number: A10-0210). All animals were cared for according to the guidelines of the Canadian Council on Animal Care (2009) and the National Farm Animal Care Council (NFAAC, 2009).

### (b) Animals, housing and diet

The experiments were conducted using 18 calves, housed at the University of British Columbia Dairy Education and Research Centre (Agassiz, BC, Canada). Calves were separated from the dam within 6 h of birth, housed in individual pens (1.2×2 m) for 4 d, and then assigned to either continued individual housing (*n* = 8) or pair housing (*n* = 10 calves) roughly alternately (calves being born close together in time being more likely to be assigned to pair housing) to balance the treatments over time. Paired calves were housed in double pens measuring 2.4×2 m. Individually housed calves had visual but not physical contact with calves in neighbouring pens. Pairs were composed of calves of the same sex born within 2 days of one another. All pens were sawdust bedded. Pasteurized whole milk was fed via teat, 4 L twice a day. Starter, hay and water were available *ad libitum*.

### (c) Experiment 1

**Figure 1:**
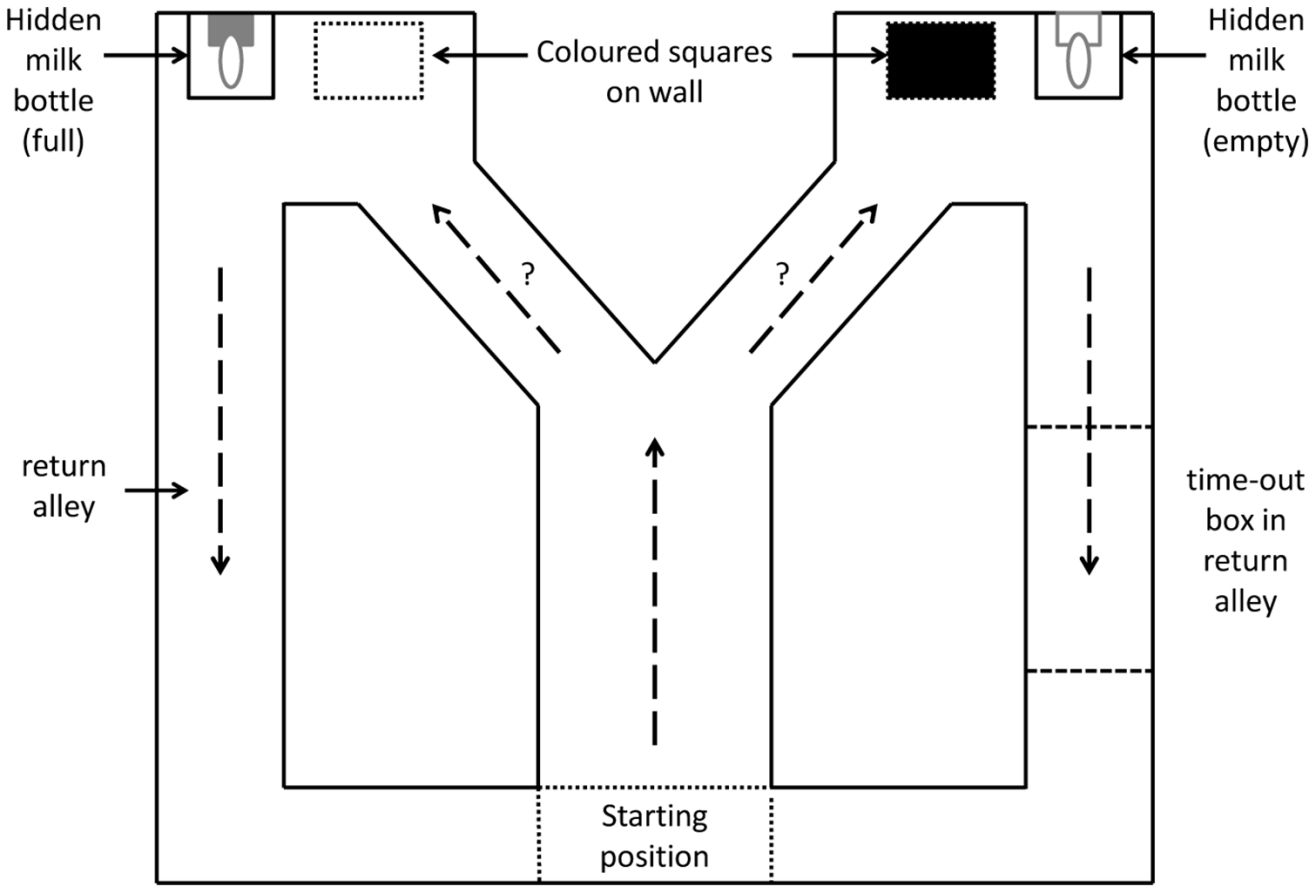
Experimental pen with Y-maze. In this example, the left arm contained a white square associated with a positive event (full milk bottle) and the right arm contained a black square associated with a negative event (empty milk bottle, followed by 20 sec in the time out box). Milk bottles were hidden by a bottle cover. Solid lines represent plywood partitions, and dashed arrows represent movement of the calf through the Y-maze. The starting position was indicated by painted lines on the pen floor. The experimental pen measured 3.0×4.6 m.

From birth to 3 weeks of age no testing was performed. During the last 5 days of the third week calves were led to the test pen containing the Y-maze (Fig. 1) for 10 min/d to habituate to the test setup. During the last 3 visits, calves were fed from bottles of milk placed in both arms of the Y-maze.

Training began at 4 weeks of age. Calves were trained twice a day from 07∶30 to 09∶30 and 15:00 to 17:00 h. A 2 L bottle covered with a white box was positioned at the end of one arm of the Y-maze, immediately adjacent to a white square (measuring 80×95 cm). An identical box, bottle and square were placed in the opposite arm but in this case the base and square were painted black. Calves were alternately assigned to training with the white positive (i.e. the white side had a bottle containing milk and the black one was empty) or black positive (the black side had milk and the white one was empty).

The first two training sessions began with four ‘forced choices’, meaning that the calves were led into the maze with only the positive arm open, followed by 12 choice trials. Every subsequent training session consisted of only the 12 choice trials. Choice trials started when the calf crossed the start line of the Y-maze and stopped when the calf touched a teat. If the calf chose the rewarded option (positive), the calf was allowed to drink for 5 s, after which it was gently guided to the exit of the Y-maze and immediately returned to the start position for a new trial. If the calf chose the side without milk, the calf was held in that side and prevented from returning to the start position for 20 s. The discriminative stimuli (e.g. black versus white squares and boxes) were alternated between the two arms of the Y-maze using Gellerman's [10] sequence (number of trials before changing sides: 1/1/2/3/3/2), selected to ensure the animal learns the visual cue rather than location (i.e. left versus right sides of the arm). Calves received their standard allotment of 4 L of milk during each test session; no other milk was provided during the day.

Training continued until the calf reached the learning criterion of a minimum of 10 correct choices out of 12 (i.e. >80%) during three consecutive sessions. Discriminative stimuli were then reversed; i.e. calves that were initially trained to associate the white with milk now had milk access paired with the black stimulus and vice versa. Testing continued with the new stimuli (and the same Gellerman sequence) until the learning criterion was again met.

### (d) Experiment 2

At 7 weeks of age, the calves were tested individually in the test pen that had previously housed the Y-maze, beginning at 07:00 h. After 5 min of habituation, the calf was removed from the pen just long enough for a novel object (a plastic red bin, 60 cm in diameter and 40 cm in height) to be placed in the centre of the experimental pen. The same person removed the calf and placed the object for all tests. The calf was then allowed 5 min to interact with the bin. This test was repeated 8 times for each calf. Repeated tests were after intervals of 10, 30, 60, 120, 360, 720 and 1440 min, requiring almost 2 d to complete. The time calves had their head oriented towards the object at a distance ≤20 cm and the time spent exploring the object (defined as sniffing, licking, and pushing the object) were measured during each test.

### (e) Statistical analysis

In Experiment 1 we recorded each choice as correct or incorrect, allowing us to calculate % correct choices per session per calf. For both the initial discrimination learning and the reversal learning phases, we calculated the number of sessions required for each calf to reach the learning criterion. Calves required a minimum of 9 sessions (108 trials) to acquire the simple discrimination and 11 sessions (132 trials) to acquire the reversal discrimination. To assess the effect of pair versus individual housing, we compared the percentage of correct choices over these sessions. The effect of the housing treatment was tested using the MIXED procedure in SAS (version 9.3), specifying an autoregressive covariance structure, with session as a repeated measure and calf as subject. Residuals were tested for normality. Session x treatment interactions were tested but were never significant. Data were analysed separately for the simple discrimination phase and the reversal phase. The number of sessions to criterion was non-normally distributed and treatments were compared using a Wilcoxon signed rank test. For practical reasons, training was not continued beyond 19 sessions for the final calves; any calves that had not reached the criterion of 80% correct by this time were assigned the value of 21 for sessions to criterion. Seventeen calves were trained but data from the first 2 pair-housed calves were discarded because of a change in the training procedure (these calves were trained with smaller panels, measuring 30×30 cm), leaving a total of 8 individual calves and 7 pair calves in the analysis.

For Experiment 2, the effect of the repeated testing sessions on time spent exploring the novel object was tested using the MIXED procedure in SAS. The model specified session number as a repeated measure and calf as subject, again using an autoregressive covariance structure. This test was performed separately for pair-housed and individually housed calves. To normalize variance, exploration times were square-root transformed before analysis. Significance was declared at P<0.05. Means are reported ± SE.

## Results

### (a) Experiment 1

**Figure 2:**
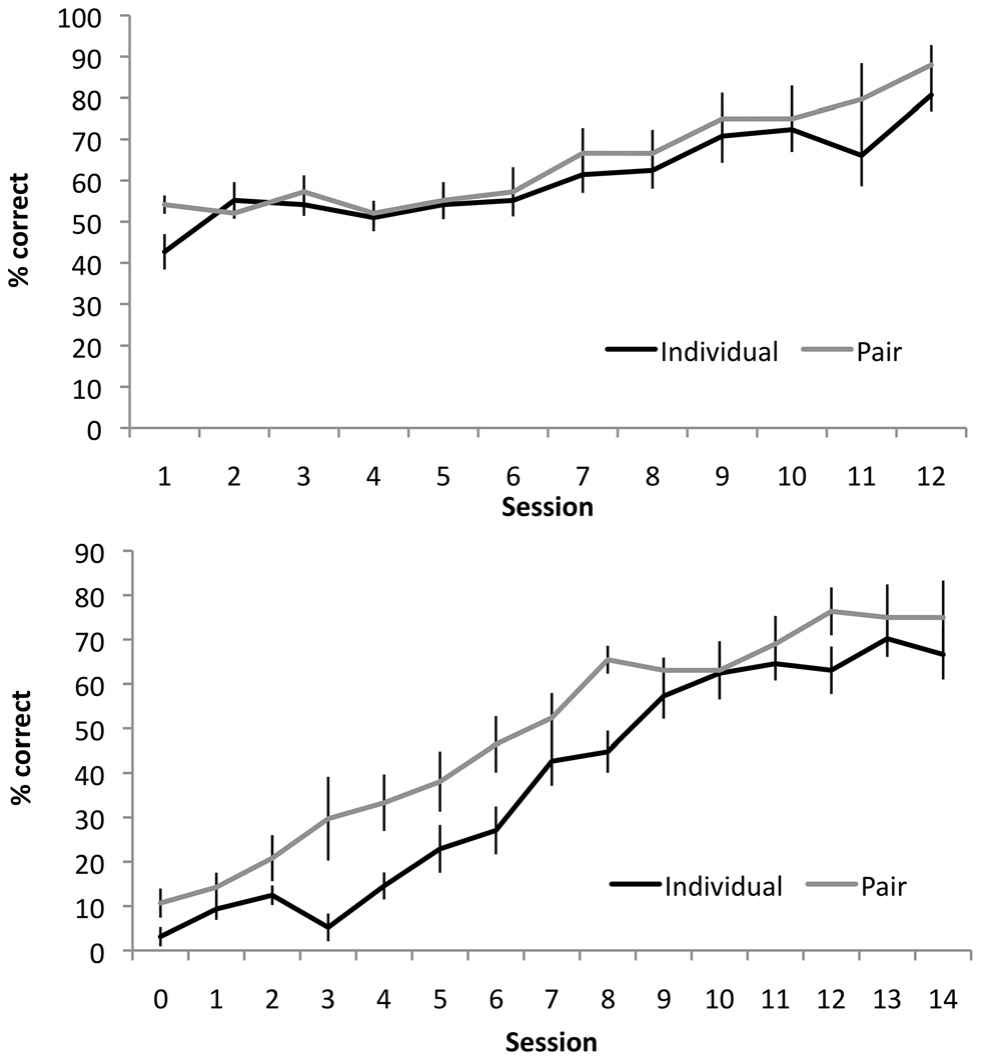
Mean ± SE correct responses for individual and paired calves during (a) the initial colour discrimination task, and (b) the reversal task. Individual (*n* = 8) and paired calves (*n* = 8) performed similarly in the initial discrimination task, but individual calves had significantly lower number of correct choices throughout the reversal learning task (*p* = 0.018) compared to paired calves (*n* = 7). Testing ended once calves reached the learning criterion of 80% correct over two sessions. The first calf reached criterion at session 11 of reversal; after 14 sessions, 4 calves had reached the criterion. For sessions 12 to 14 the averages displayed were calculated using the calves still undergoing testing and the mean performance the last two complete sessions of calves that had reached the criterion and were no longer being tested.

During the initial discrimination training, calf performance (% correct) improved over repeated training sessions with no effect of individual versus pair housing (Fig. 2a; F_1,14_ = 0.47, *p* = 0.49). Individual calves reached the learning criterion after a median of 15 training sessions (interquartile range 12–15) and pair calves at 12 (10–15) sessions (Z = −1.10, *p* = 0.27). When the training stimuli were reversed, calves in both treatments performed poorly. Initially during the first few sessions, all calves performed poorly, with performance below chance (50%), as calves persisted in responding as if the reinforcement had not been reversed. However, pair-housed calves then began to respond to the rewarded option while individually housed calves continued to do poorly such that the overall number of correct choices during reversal training was lower for the individually-housed calves than pair-housed calves training (Fig. 2b; F_1,14_ = 7.33, *p* = 0.018). Calves from both treatments eventually reached the learning criterion; the median number of sessions needed to reach this criterion was 19.5 (interquartile range: 15.3–21) for the individually housed calves versus 13.0 (12–21) for the paired calves (Z = −0.94, *p* = 0.34).

### (b) Experiment 2

**Figure 3:**
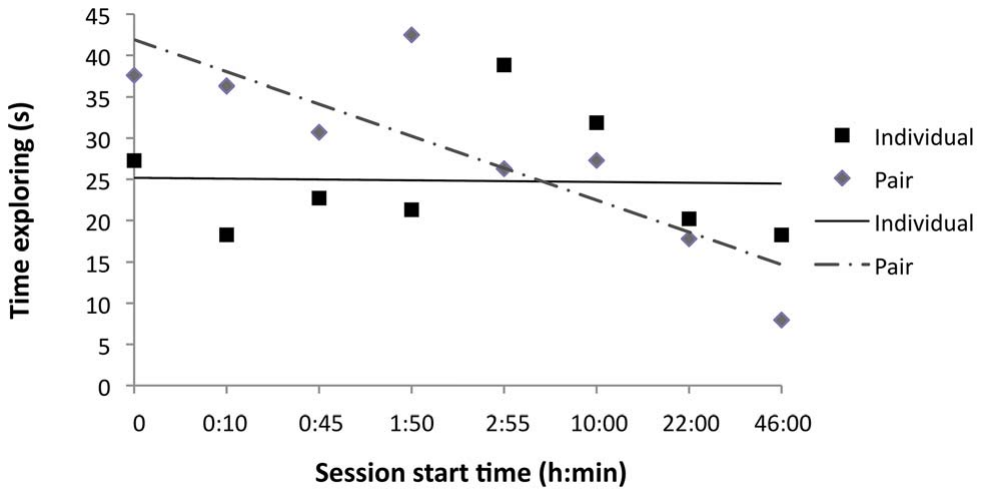
Mean time spent exploring novel object for individual and paired calves. Calves were exposed to a novel object over 8 sessions and time spent exploring was recorded (back-transformed from square-root). Exploration was defined as sniffing, licking, and pushing the object. Pair-housed calves (*n* = 10) showed a decline in exploration time over repeated sessions separated by increasing time intervals (*p* = 0.032); individual calves (*n* = 8) showed no change.

Housing treatment did not influence time spent exploring the object in the first session (t = −0.18, d.f. 11, *p*>0.10), which averaged 55.4±22.2 s for individual calves and 50.9±12.2 s for pair calves. Individually-housed calves showed no significant decline in time spent exploring the object over repeated sessions (Fig. 3; F_1,55_ = 0.08, *p*>0.10), but the pair-housed calves significantly reduced their time spent exploring with repeated testing (F_1,69_ = 4.74, *p* = 0.033).

## Discussion

### (a) Experiment 1

As expected, calves housed individually made more errors in the reversal task than did calves housed in pairs, suggesting that social isolation during this period impaired this form of learning. This result is consistent with those of De Paula Vieira and colleagues [5], who found that early social contact reduced the time calves required to use a new feeder, a difference these authors attributed to a cognitive deficit in individually housed calves. These results also correspond to those from the rodent literature: isolate-reared rats typically show impaired performance in reversal learning tests [11], [12]. In the present experiment we found no difference in the number of sessions to criterion, but this may have been due to a lack of power.

As in rodents, social isolation early in life produced significant deficits only in certain forms of learning: differences were detected only in the reversal phase and not in initial discrimination learning. Such deficits in reversal learning or other tasks that involve changing response contingencies are considered to indicate a lack of behavioural flexibility; that is, the inability to alter behaviour in response to environmental stimuli (e.g. [13]). At a behavioural level, inflexibility in the individually-reared animals can be explained as the result of living in a more predictable environment; social contact introduces variability into the environment (e.g. [14], [15]), and animals that are reared without this complexity may be less able to cope with it [16], [17]. Captivity in general tends to induce decreased flexibility [18]. Work on rodents has identified neurobiological differences associated with specific forms of behavioural inflexibility (e.g. [13]). For example, lesions to the orbital prefrontal cortex impair reversal learning, which involves a simple change in contingency, whereas responding to shifts in more abstract rules seems to depend on the medial prefrontal cortex [19]. Pathways between the hippocampus and the prefrontal cortex are also involved in the ability to inhibit behaviour, which is one component of responding appropriately to shifting contingencies [20]. We suggest that individual housing in dairy calves impairs the development of these neural structures. Similar deficits in flexibility are sometimes observed in animals that exhibit abnormal behaviours (see [21], [22]), frequently observed in animals that have lived in environments that were somehow suboptimal [23].

### (b) Experiment 2

As predicted, calves housed in pairs habituated to the novel object over repeated testing sessions, indicating that they learned to recognize it. There was no evidence of such learning in the calves housed individually. The mechanism by which habituation is either prevented or slowed in isolation-reared calves is unclear. This finding could be interpreted as further evidence of a cognitive deficit, perhaps the result of lasting brain dysfunction; responses to a novel object are an accepted measure of cognition, particularly memory [24], [25].

The lack of habituation to the novel object could also be due to psychological states induced by the isolation housing, such as the increased anxiety (reflected by behaviour in tests such as the elevated plus maze and avoidance of novel foods) and increased sensation-seeking motivation reported in isolation-reared rodents (reviewed by [6]). Previous work has frequently made use of novel object tests to assess differences in fearfulness (e.g. [26]). Other tests involving exposure to novelty have indicated that social housing affects fear in calves. For example, Jensen and colleagues [1] compared the responses of individually and group-housed calves to a novel arena, and found that individually reared calves were more fearful, as indicated by heart rate responses and less time spent in the centre of the arena. Individually-housed calves are also more fearful when first exposed to a new social partner [3]. Together these earlier studies suggest that individually-housed calves are more fearful of novelty, although in the present experiment there was no significant difference between the two housing types in how much time the calves spent interacting with the novel object during the first testing session. Anxiety might delay habituation by preventing the calves from approaching and thus learning to recognize the novel object or by making them slower to consider it non-threatening. Prolonged interest in the object could also be a form of sensation seeking indicative of boredom-like states caused by environments lacking in stimulation [27], [28]. Anxiety seems to be the more parsimonious of these two explanations, given that it could also lead to impairments in the learning task (see below).

Calves were tested using a range of inter-trial intervals because longer periods between trials were expected to interfere with recognition. Moreover, work with rats has shown that isolates had difficulties with recognition at shorter intervals than socially reared animals [8]. Our design did not allow us to separate the effects of number of exposures and interval length, but it is clear from the results that an increasing interval between sessions did not result in increasing exploration (see Fig. 3) as would be predicted if more individuals forgot their previous experiences with the object. However, future work should test the effects of interval length independent of repeated testing.

### (c) General discussion

The results of these two experiments suggest that individual rearing results in cognitive impairments in dairy calves. Future experiments should investigate the role of emotional states on these apparent learning deficits. For example, fear and anxiety are known to impair learning in humans [30] and other animals [29]; these effects could be tested by providing some animals with an anxiolytic before testing. Differences in sensation-seeking could be investigated by presenting calves with a range of objects expected to differ in valence: individually-housed calves might be expected to explore whatever stimuli are offered if they are experiencing boredom-like states, even if those stimuli are familiar (when novel stimuli are not available) or would typically be frightening (cf. [27]).

The observed impairment in isolation-reared calves is a concern for welfare regardless of whether it is related to anxiety or boredom. Some have argued that cognitive ability is not necessarily tightly linked to welfare under captive conditions because under stable situations habitual responses may be adequate and take less time and energy than more flexible responses (e.g. [29], [31]). However, dairy cattle are faced with many challenges as part of their routine management, including changes in feeding environment, social regroupings and interacting with new technologies including robotic milking equipment and automated feeders. Individuals that are more flexible might adapt more quickly to these changes, improving the lives of the animals and the farmers that work with them.

We do not yet know the extent of the cognitive deficits produced by individual rearing. Reversal learning tests are just one way we can assess an animal's flexibility in learning, and performance in these tasks is potentially affected by a variety of different neurological changes [32]. Other tasks such as extra-dimensional set-shifts (in which the animal must shift attention to a different sensory quality or “dimension” of the stimulus) are also considered measures of flexibility but involve different neural structures [19] and may be differentially affected by the environment as they are by some forms of stress [33]. These types of set-shifts could be used to determine whether the deficits seen here are signs of a more general cognitive impairment or are due to changes in the development of one specific neural region. Based on the work with rats reared socially or in isolation, the impairments seen here are expected to extend to extra-dimensional set-shifting [8], [34], [35]. Because these tasks are more challenging [36], [37], they may be more sensitive for detecting differences in the number of sessions to criterion.

All calves were socially isolated during cognitive testing and this may have differentially affected performance. For example, the more rapid habituation of paired calves in the novel object test may have reflected a higher motivation to leave the pen and return to their social partner. However, we would expect that the pair-reared calves would experience more acute stress (associated with separation from the pen mate) than would the individually reared calves, reducing success in the reversal task. Since we found the opposite (i.e. poorer performance by the individually housed calves), it seems likely that the present results reflect more lasting differences in cognitive abilities rather than a temporary effect of testing conditions.

## Conclusion

Calves were able to learn a simple colour discrimination task, and then re-learn the task when the colour treatments were reversed. The speed of learning for the simple discrimination task was similar for individually housed and pair-housed calves, but the pair-housed calves were able to adapt more easily when the training stimuli were reversed. Pair housed calves also showed evidence of learning to recognize a novel object, but individually housed calves failed to habituate over the course of the experiment. Together, the results of these studies suggest that individual housing of dairy calves can result in measurable learning deficits. Social housing for calves may result in animals that are more flexible in their responses to changes in management and housing.
